# Hard Pill to Swallow: A Recurrent Zenker-like Diverticulum

**DOI:** 10.14309/crj.0000000000001063

**Published:** 2023-07-13

**Authors:** Kinnari Modi, Chiemeziem Eke, David Lee, Paul Tarnasky

**Affiliations:** 1 Internal Medicine, Methodist Dallas Medical Center, Dallas, TX; 2 Gastroenterology and Hepatology, Methodist Dallas Medical Center, Dallas, TX; 3 Methodist Digestive Institute, Methodist Dallas Medical Center, Dallas, TX

## CASE REPORT

**Figure 1.**
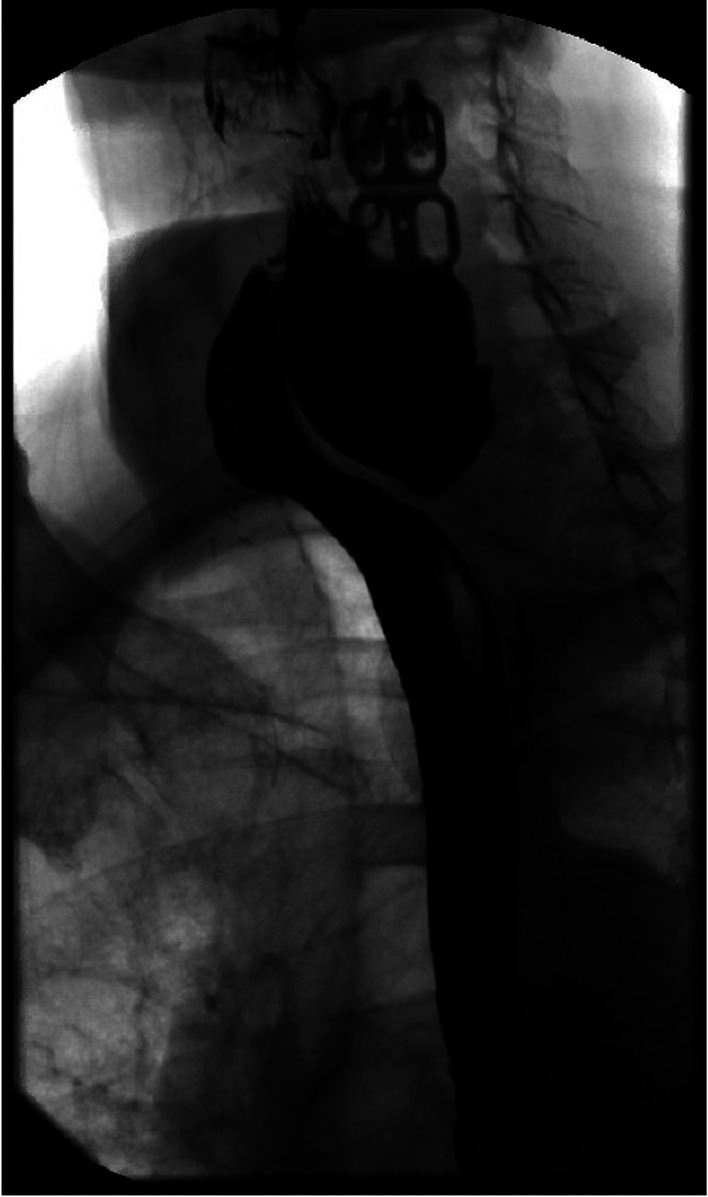
Large left lateral diverticulum of the cervical esophagus without perforation.

**Figure 2.**
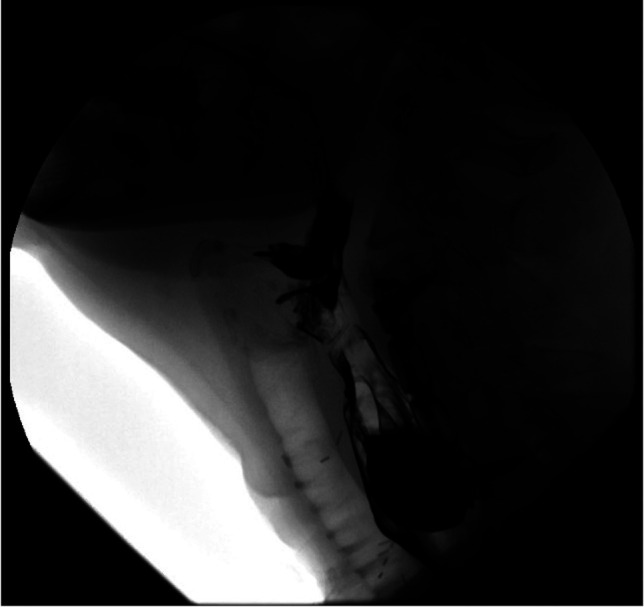
Cervical hardware in close proximity to the diverticulum.

**Figure 3.**
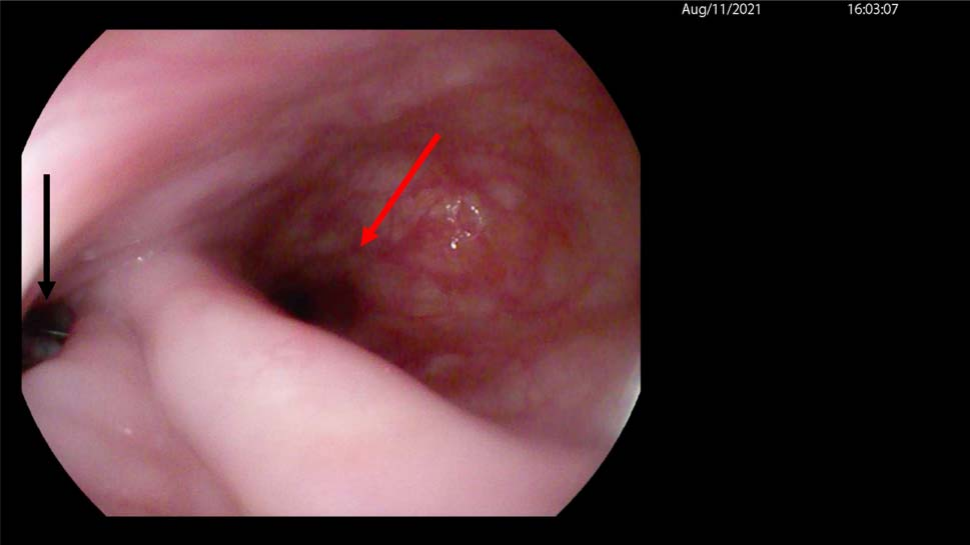
Red arrow: True lumen, black arrow: false lumen (diverticulum).

**Figure 4.**
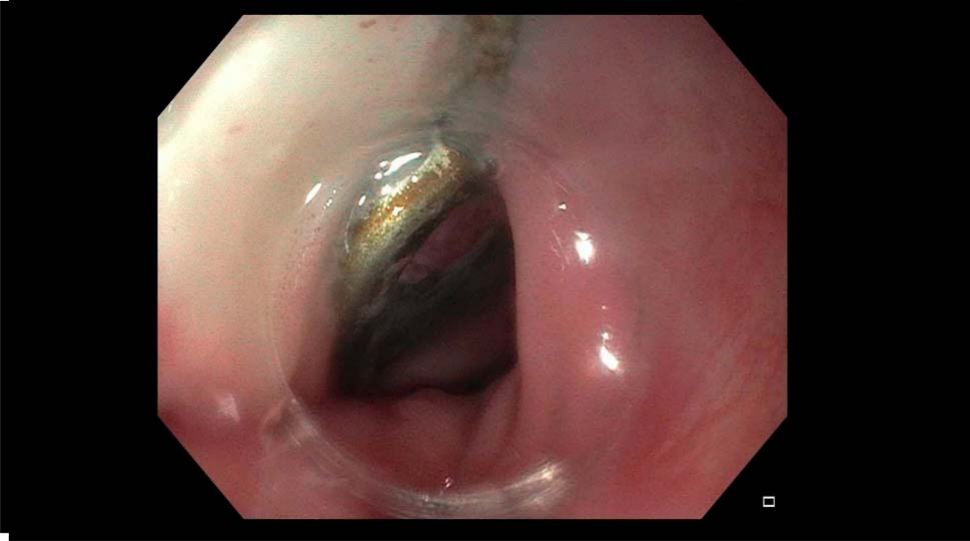
Cervical spine fixation plate eroding through the mucosa of the diverticulum.

Zenker diverticulum (ZD) is an uncommon disease mostly of the elderly male population. Other types of pharyngoesophageal diverticula have also been reported as a rare complication of anterior cervical spine surgery.^1,2^ This is a case of a 63-year-old woman who presented with complaint of recurrent dysphagia and a 15 pound weight loss over the past 2 months. Her medical history is pertinent for C3-C6 anterior cervical fusion 19 years before and surgical myotomy 12 years ago for reported ZD that presented with similar symptoms. Barium esophagram showed a left lateral diverticulum of the cervical esophagus without serosal perforation and cervical hardware near it (Figures 1 and 2). During upper endoscopy, the gastroscope intubated the true esophageal lumen rather than the false lumen created by the diverticulum (Figure 3). Her cervical spine fixation plate had eroded through the mucosa of the diverticulum (Figure 4). Once this was visualized, the endoscopy was aborted, and she was referred for surgical removal of the hardware and diverticulectomy. Given her young age and existing hardware at the time of reported ZD diagnosis several years before, she likely had an atypical traction diverticulum linked to the cervical hardware with recurrence of this after surgical myotomy.

## DISCLOSURES

Author contributions: K. Modi: review of literature, substantial contributions to the conception and design of the work, and drafting and revising the work. C. Eke: substantial contributions of the conception and design of the work and drafting and revising work. D. Lee, P. Tarnasky: analysis and interpretation of data for the work and revising the work critically for important intellectual content. K. Modi is the article guarantor.

Financial disclosure: None to report.

Informed consent was obtained for this case report.
